# Microalbuminuria among Newly Diagnosed Diabetic Patients at Mulago National Referral Hospital in Uganda: A Cross Sectional Study

**DOI:** 10.23937/2572-4010.1510021

**Published:** 2018-08-23

**Authors:** Muddu Martin, Mutebi Edrisa, Isaac SSinabulya, Kizito Samuel, Mulindwa Frank, Mondo Charles Kiiza

**Affiliations:** 1 Department of Medicine, Makerere University College of Health Sciences, Mulago Hospital Complex, Uganda; 2 Clinical Epidemiology Unit, Makerere University College of Health Sciences, Uganda

## Abstract

**Background:**

Microalbuminuria is an early marker of nephropathy, cardiovascular diseases and severe ocular morbidity in adults with diabetes mellitus. This subclinical condition is associated with high morbidity and mortality. Microalbuminuria precedes the development of overt diabetic nephropathy by 10–14 years. At this stage, one can reverse diabetic nephropathy or prevent its progression. Unfortunately, tests to detect microalbuminuria in diabetics are not routinely done in Uganda. This study sought to determine the prevalence and factors associated with microalbuminuria among newly diagnosed diabetic patients in the National Referral Hospital in Uganda.

**Methods:**

In this cross-sectional study conducted between June 2014 and January 2015, we recruited 175 newly diagnosed adult diabetic patients. Information on patients’ socio-demographics, biophysical profile, blood pressure measurement, biochemical testing and echocardiographic findings was obtained for all the participants using a pre-tested questionnaire. Microalbuminuria was defined as Albumin to Creatinine Ratio (ACR) between 30 and 299 mg/g. Bivariate and multivariate logistic regression analyses were used to investigate the association of several factors with microalbuminuria.

**Results:**

Of the 175 patients recruited, males were 90 (51.4%) and the mean age was 46 ± 15 years. Majority of patients had type 2 DM 140 (80.0%) and the rest had type 1 DM 35 (20.0%). The mean HbA1C was 13.9 ± 5.3%. Mean duration of diabetes was 2 months. Prevalence of microalbuminuria was 47.4% (95% CI: 40.0%−54.9%) among all the patients that were assessed in the study. The independent factor associated with microalbuminuria was pregnancy (OR7.74[95% CI: 1.01–76.47] P = 0.050) while mild and moderate physical activity at work were inversely associated with microalbuminuria respectively (OR0.08[95% CI: 0.01–0.95] P = 0.046) and (OR0.07[95% CI: 0.01–0.77] P = 0.030).

**Conclusions:**

Prevalence of microalbuminuria was high in this patient population of newly diagnosed diabetes mellitus. Pregnancy was positively associated with significant microalbuminuria while physical activity at work was inversely associated with microalbuminuria. Early detection and management of microalbuminuria in asymptomatic individuals may help in preventing deterioration in renal function and development of overt diabetic nephropathy and progression to ESRD.

## Background

Microalbuminuria is an early marker of nephropathy, cardiovascular diseases and severe ocular morbidity in adults with diabetes [1–5]. It is a subclinical condition that is associated with high morbidity and mortality [5,6]. Diabetes mellitus is one of the leading causes of microalbuminuria in SSA [5,7,8].

The presence of microalbuminuria precedes the development of overt diabetic nephropathy by 10–14 years. It is at this stage that one can reverse diabetic nephropathy or prevent its progression [5,8–10]. Unfortunately, tests to detect microalbuminuria in diabetics are not routinely done in Uganda and SSA as a whole.

Among persons with DM, microalbuminuria has been estimated to be twice the prevalence in the general population in Africa [5,11,12]. Approximately half the patients with microalbuminuria will progress to overt proteinuria over the next decade [6,13]. Therefore, early detection and appropriate interventions in asymptomatic individuals may help in preventing deterioration in renal function and development of overt diabetic nephropathy and progression to ESRD [5].

Therapeutic interventions which reverse microalbuminuria include intensified glycemic control, use of ACE inhibitors and these should be initiated in diabetics with microalbuminuria to prevent progress to overt diabetic nephropathy [12].

Diabetic nephropathy, the end result of microalbuminuria, is a major cause of morbidity, premature mortality, end stage renal disease, need for renal replacement therapy, cardiovascular diseases, and escalating health-care costs in diabetic patients [6,12,14–18]. The prevalence of DN is increasing steeply along with the diabetes epidemic [15]. Approximately one third to half of patients with diabetes develops renal manifestations [14,15,19].

DN is more frequent among patients in Africa as compared to those in the developed world due to delayed diagnosis, limited screening and diagnostic resources, poor glycemic control and inadequate treatment of microalbuminuria [14,19,20].

From studies in the western world, the factors associated with microalbuminuria include obesity, hypertension, diabetes mellitus, poor glycemic control, body mass index, increased duration of diabetes, proliferative retinopathy, peripheral neuropathy, age and BMI [5,6,12]. In Africa, there is paucity of data on the prevalence of microalbuminuria and diabetic nephropathy among diabetic patients [5,14].

Therefore, this study sought to determine the prevalence and factors associated with microalbuminuria among newly diagnosed diabetic patients at a National Referral Hospital in Uganda.

## Methods

### Study design and participants

This was a cross-sectional study among 175 newly diagnosed diabetic patients at Mulago National referral hospital in Uganda conducted between June 2014 and January 2015. All newly diagnosed diabetic patients aged 18 years and above attending the diabetic clinic or admitted to the medical wards of Mulago hospital during the study period who met the inclusion criteria and provided informed consent were recruited consecutively. We excluded patients with urinary tract infection and patients who were unable to provide information.

### Study setting

The study was carried out in the diabetic outpatient clinic, the medical endocrine ward and the medical emergency ward of Mulago National referral Hospital. Mulago is the only National referral hospital for Uganda and Teaching Hospital for Makerere University with a bed capacity of 1500. Mulago hospital receives referrals from all parts of the country including referrals from neighboring countries like Southern Sudan, the Democratic Republic of Congo, Rwanda among others. The study population is representative of the Ugandan diabetic population.

### Sample size estimation

Using prevalence of 17% for microalbuminuria among diabetic patients as determined by Son MK, et al. [1], a sample size of **180** was estimated using the Kish Leslie (1965) formula.

$$\text{N}=\frac{{\text{Z}}^{2}\text{P}(1-\text{P})}{{\text{D}}^{2}}$$

N = Sample size **180**

Z = 1.96, the normal value corresponding to the 95% confidence interval

P = 0.17, prevalence from the above study

D = 0.05, Acceptance error

However, data from 5 participants was not included because it was incomplete. Therefore, we analyzed data from **175** participants.

### Clinical assessment

We took a focused history and performed a specific physical exam to determine the biophysical measurements. Information gathered was entered into a **pre-tested** questionnaire. We aassessed the following factors: patients’ demographic data, history of hypertension, age, physical exercise, marital status, date of diagnosis of DM, drug history, occupation, education level, and LNMP. We classified physical activity according to the World Health Organization, Global Strategy on Diet, Physical Activity and Health [21].

Weight was measured using a Secco weighing scale to the nearest Kg, height was measured in meters using a non-stretchable tape and these were used to compute Body Mass Index (BMI). Waist and hip circumferences were measured and waist to hip ratios determined for all patients.

Glycated haemoglobin HbA1C was measured by automated high-performance liquid chromatography.Other investigations included urinalysis and Microalbuminuria using ACR.

Echocardiography parameters were acquired using a commercially available machine, Phillips HD11XE (Eindhoven, The Netherlands) with 2-D, MMode and Doppler capabilities was used according to the American Society of Echocardiography [22].

All equipment used were calibrated to avoid measurement bias.

### Assessment of microalbuminuria

We explained to participants the procedure for collecting a midstream urine sample. Each participant was given two urine containers and instructed to provide 2 separate samples of midstream urine each measuring 10 ml. One of the containers was sterile and the sterile sample was used for urinalysis including urine microscopy. We excluded participants who were found to have urinary tract infection and we started them on antibiotics. The second container collected a spot urine sample for measurement of urine albumin to creatinine ratio (ACR). Microalbuminuria was defined as ACR between 30 and 299 mg/g.

### Blood pressure measurement

Blood pressure was measured using a mercury sphygmomanometer and an average of the 2 readings was used in the analysis.

We instructed each participant to sit on a chair with both feet resting on the floor uncrossed and rest for 5 minutes before taking the 2 blood pressure readings. The two blood pressure readings were recorded 5 minutes apart using a standard manual deksametazon MK3 mercury sphygmomanometer.

The patient exposed the arm from which blood pressure was to be measured and rested it on a table at the level of the heart. A blood pressure cuff (with a bladder length > 80% of the arm circumference) was placed 2 cm above the antecubital fossa. We inflated the cuff while feeling for the patient’s radial pulse.

The estimate of the systolic blood pressure was obtained by inflating the cuff 30 mmHg above the point of disappearance of the radial pulse. We then placed the diaphragm of the stethoscope on the antecubital fossa and the cuff deflated at 2 mmHg per second while listening for korotkoff sounds.

Korotkoff sounds 1 and 2 represented systolic and diastolic blood pressures respectively. For cases where Korotkoff sounds remained audible despite complete deflation of the cuff, abrupt muffling of the sounds was used to denote diastolic blood pressure. We recorded the average of the 2 blood pressure readings. Participants were declared hypertensive if they; were on anti-hypertensive medication, had history of hypertension and/or evidence of hypertension (blood pressure ≥ 140/90 mmHg).

### Ethical approval

Institutional consent was sought from the department of Medicine Makerere University, Mulago National Referral Hospital and School of Medicine Research and Ethics Committee (SOMREC) of Makerere University College of Health Sciences. All study participants provided written informed consent for involvement in the study. Enrolment was totally free and voluntary and participants were free to withdraw at any time without any consequences to them. The patients’ Records/information was anonymized and de-identified prior to analysis.

### Data management and analysis

Data was double entered in a database developed with EPIDATA version 3.1, validated and inconsistences cleared then exported to STATA 13 for analysis. Continuous data was summarized using measures of central tendency while categorical data was summarized as frequencies and percentages and presented in tables. Prevalence was presented as percentages with their confidence intervals. Comparisons were made using the student t-test for the continuous data and Chi-square or fisher’s exact test for the categorical data.

The outcome was dichotomized as having microalbuminuria or not then logistic regression was used to determine the association between the predictors and microalbuminuria. This was presented as Odds ratio (OR) and their 95% confidence interval (CI). Only factors with P- value < 0.2 at bivariate analysis were considered for multivariate analysis. Multivariate logistic regression was performed; interaction was assessed for using the Chunk test. Confounding was assessed for using a 10% difference between the crude and adjusted models. Significance was at p value of 0.05 and less.

## Results

### Characteristics of newly diagnosed diabetic patients at Mulago National referral hospital who participated in the study

This study recruited 175 newly diagnosed diabetic patients between June 2014 and January 2015. Of these, 90 (51.4%) were males. The mean age of all the participants was 46 ± 15 years. Majority of patients had type 2 DM 140 (80.0%) and the rest had type 1 DM 35 (20.0%). The mean HbA1C was 13.9 ± 5.3%. Mean duration of diabetes was 2 months (Table 1 and Table 2).

### Prevalence of microalbuminuria

Prevalence of microalbuminuria was 47.4% (95% CI: 40.0%−54.9%) among all the patients that were assessed in the study. Among these patients, male participants had a higher prevalence of microalbuminuria of 51.1% compared to females with 43.5%. Type 1 DM patients had a higher prevalence of Microalbuminuria 19 (54.3%) compared to 64 (45.7%) among type 2 DM. Patients with central obesity as measured by waist hip ratio had a higher prevalence of Microalbuminuria of 57.7% compared to those without central obesity whose prevalence was 49.6% Table 2.

### Factors associated with microalbuminuria

In bivariate analysis, the factors associated with microalbuminuria included: being overweight and obesity. (Refer to Table 3).

After adjusting for patients’ sex, age, hypertension, anti-hypertensive drugs and level of HbA1C, the only significant factor associated with microalbuminuria was pregnancy (OR7.74[95% CI: 1.01–76.47] P = 0.050) while mild and moderate physical activity at work were inversely associated with microalbuminuria respectively (OR0.08[95% CI: 0.01–0.95] P = 0.046) and (OR0.07[95% CI: 0.01–0.77] P = 0.030). Overweight and obesity has no significant association with microalbuminuria. (Refer to Table 3).

## Discussion

In this study we found a high prevalence of microalbuminuria among the group of diabetics who were newly diagnosed with the disease. Approximately half of all participants had significant microalbuminuria 47.4%. Prevalence of microalbuminuria among type 1 diabetics was 54.3 while type 2 diabetics had 45.7%. The prevalence is rather higher than what Mi Kyung, et al. found. In their population, type 1 diabetic patients had prevalence of Microalbuminuria of 17%. Mi, et al. assessed adolescents with median age of 18.9 years while our population had a mean age of 46 ± 15 years, the effect of age could explain the difference.

Among type 2 diabetic patients, we found a prevalence of 45.7% which is comparable to findings by Mi Kyung, et al. they had a prevalence of microalbuminuria of 44.4% among type 2 diabetic patients [1]. This high prevalence may be due to a period of latency for patients with type 2 DM before diagnosis.

Alleyn noted that approximately one third of persons with type 1 DM develop microalbuminuria [6,18,23]. The prevalence we found was slightly higher that Alleyn’s estimate possibly because of advanced age for our patient population compared to their population of adolescents. Chowta noted that in type-2 diabetics, microalbuminuria ranges from 8–47% [12,24,25]. These findings are in agreement with our findings for patients with type 2 DM. Jean Jacques added more evidence to the fact that approximately one third to half of patients with DM develops renal complications [14,26].

In our patient population, age had no effect on the degree of MA and Lampropoulou reached a similar conclusion in their study where age showed just a weak correlation with MA [6,27]. Although microalbuminuria was more common in participants 40 years and older in our study, this is predictable because age and diabetes duration for more than 10 years are well known risk factors for the development of diabetic nephropathy [16].

We found the prevalence of microalbuminuria to be more common among females than males and Okpere, et al. came to a similar conclusion in their study of Nigerian adolescents. Similarly, patients who had central obesity in our study had a higher prevalence of MA compared to those without central obesity, Okpere reports similar findings [5].

The prevalence of microalbuminuria we found of 47.4% is 3 times higher than that of the general population which is reported to be 10–15% [5]. However most of this evidence is from the developed world and in Africa, there is paucity of data on the prevalence of microalbuminuria both in the general population and among diabetics [5].

Among diabetic patients, microalbuminuria predicts the development of overt diabetic nephropathy [5]. Therefore, a high prevalence we found predicts a great burden of renal disease for our patient population if no interventions are done.

Individuals with microalbuminuria may rapidly progress to overt proteinuria, renal dysfunction and end stage renal disease (ESRD) later in life. Therefore, for primary prevention of ESRD, nephrologists and physicians should aim at early detection of microalbuminuria in asymptomatic individuals, so that appropriate interventions can be commenced early [5,8,9].

In our study, microalbuminuria had no significant association with BMI and this is in keeping with finding by Chowta, et al. who found that there is no effect of BMI and sex on the prevalence of microalbuminuria [12]. Likewise, sex had no significant association with microalbuminuria in this study. Patients who exercised mildly and moderately during work were less likely to develop microalbuminuria. This makes exercise a possible protective factor against microalbuminuria among diabetics.

Pregnant patients in our study were seven times more likely to have significant microalbuminuria compared with those who were not pregnant. However, the association is marginally significant as the range of confidence interval is considerably wide owing to the small sample size. Moreover, the presence of microalbuminuria during pregnancy could be attributed to gestational diabetes that may resolve after giving birth. Other possible reasons could be the pregnancy associated protein loss and likely hypertension in pregnancy. These are potential confounders in our study.

In our study there was no association of microalbuminuria with glycemic control as measured by HbA1C. This is different from findings by Alleyn, et al. In their study Microalbuminuria occurred in association with poor glycemic control and elevated blood pressure [6]. In addition, hypertension had no significant association with microalbuminuria in this study which is in contrast with Alleyn’s findings.

The differences could be due to the differences in age for the two populations. Alleyn, et al. assessed a predominantly young population of type 1 Diabetics while we assessed a population with mean age of 46 ± 15 years. In addition, Alleyn, et al. followed up their cohort for 2 years yet ours was a cross sectional study [6].

Gender was not associated with microalbuminuria in this study although the prevalence of microalbuminuria was higher among females than males; this difference was not statistically significant. Chowta, et al. reported similar findings: There was no correlation between gender and microalbuminuria in type-2 diabetes mellitus [12,28].

Studies in the Western world show a positive correlation between degree of microalbuminuria, BMI and blood pressure [12]; these findings are different from evidence generated in our study where there is no association of microalbuminuria with hypertension and BMI.

Microalbuminuria precedes overt diabetic nephropathy by 10–14 years and at this stage one can reverse diabetic nephropathy or prevent its progression. Therapeutic interventions which reverse microalbuminuria include intensified glycemic control, use of ACE inhibitors and blood pressure control.

Evidence shows that DN is more frequent among patients in Africa as compared to those in the developed world. Possible reasons include delay in diagnosis, limited screening/diagnostic resources, poor control of blood sugar and blood pressure and inadequate treatment at an early stage [14,19,20]. However, evidence on the burden of kidney diseases in people with diabetes in Africa remains very patchy [14].

We recognize that the lack of a non-diabetic control population is a limitation of our study; however, this study was not designed to determine the effect of diabetes on the kidney, but rather to explore the magnitude of subclinical microalbuminuria among newly diagnosed diabetic patients in the SSA context, including the factors associated with microalbuminuria.

The short duration of the study could have obscured seasonal variability. This was due to limitations in logistics: however newly diagnosed diabetic patients were recruited consecutively over a seven months period.

We did not analyze data from 5 participants because it was incomplete. This limitation could have affected the power of the study.

## Conclusion

Prevalence of microalbuminuria was high in this patient population of newly diagnosed diabetes mellitus. Pregnancy was positively associated with significant microalbuminuria while physical activity at work was inversely associated with microalbuminuria. These findings suggest that healthcare policy or research efforts may focus on reversing microalbuminuria in hopes of improving the prognosis of diabetic patients.

## Figures and Tables

**Table 1:** Social demographic characteristics of newly diagnosed diabetic patients at Mulago National referral hospital who participated in the study (N = 175).

Characteristic		Total (N)	Microalbuminuric N (%)	Normoalbuminuric N (%)
Age	< 40 years	50 (28.5)	25 (50.0)	25 (50.0)
	40 years and above	125 (71.5)	58 (46.4)	67 (53.6)
Gender	Male	90 (51.4)	46 (31.1)	44 (48.9)
	Female	85 (48.6)	37 (43.5)	48 (56.5)
Employment	Employed	66 (37.7)	31 (47.0)	35 (53.0)
	Unemployed	108 (62.3)	51 (47.2)	57 (52.8)
Pregnancy	Yes	6(6.3)	5 (83.3)	1 (16.7)
	No	90 (93.7)	38 (42.2)	52 (57.8)
Education	None	14 (8.5)	7 (50.0)	7 (50.0)
	Primary	71 (6.6)	33 (46.5)	38 (53.5)
	Secondary	64 (36.6)	29 (45.3)	35 (54.7)
	Tertiary	26 (14.9)	14 (53.9)	12 (46.2)
Marital status	Never married	26 (14.9)	14 (53.9)	12 (46.1)
	Currently married	104 (59.4)	48 (46.1)	56 (53.9)
	No longer married	45 (25.7)	21 (46.7)	24 (53.3)

**Table 2:** Characteristics of newly diagnosed diabetic patients at Mulago National referral hospital who participated in the study (N = 175).

Characteristic		Total (%)	Microalbuminuric N (%)	Normoalbuminuric N (%)
Physical activity at work	Sedentary	17 (9.8)	11 (64.7)	6 (35.3)
	Mild	48 (27.7)	23 (47.9)	25 (52.1)
	Moderate	71 (41.0)	33 (46.5)	38 (53.5)
	Strenuous	37 (21.4)	15 (40.5)	22 (59.5)
Physical activity at leisure	Sedentary	123 (70.3)	60 (48.8)	63 (51.2)
	Moderate	52 (29.7)	23 (44.2)	29 (55.8)
DM type	Type 1	35 (20.0)	19 (54.3)	16 (45.7)
	Type 2	140 (80.0)	64 (45.7)	76 (54.3)
Hypertension	Hypertensive	108 (61.7)	47 (43.5)	61 (56.5)
	Normotensive	67 (38.3)	36 (53.7)	31 (46.3)
BMI (kg/m^2^)	Underweight (< 19.0)	33 (18.9)	23 (69.7)	10 (30.3)
	Normal weight (19.0 - < 25.0)	70 (40.0)	35 (50.0)	35 (50.0)
	Over weight (25.0 - < 30.0)	42 (24.0)	13 (31.0)	29 (69.0)
	Obesity (> 30.0)	30 (17.1)	12 (40.0)	18 (60.0)
Waist hip ratio	Normal < 0.90 (males) and < 0.85 (females)	123 (70.3)	61 (49.6)	62 (50.4)
	Abnormal > 0.90 (males) and > 0.85 (females)	52 (29.7)	30 (57.7)	22 (42.3)
HbA1C%	< 7%	14 (8.1)	3 (21.4)	11 (78.6)
	> 7%	158 (91.9)	79 (50.0)	79 (50.0)
Drugs	ACEI/ARBs	9 (5.1)	4 (44.4)	5 (55.6)
	CCB	12 (6.9)	3 (25.0)	9 (75.0)
	Beta blockers	4(2.3)	2 (50.0)	2 (50.0)
Ejection fraction %	> 50%	139 (79.5)	65 (46.8)	74 (53.2)
	< 50%	36 (20.5)	18 (50.0)	18 (50.0)
LVH	Present	98 (56.0)	42 (42.9)	56 (57.1)
	Absent	77 (44.0)	41 (53.3)	36 (46.7)
Diastolic function	Normal	83 (47.4)	43 (51.8)	40 (48.2)
	Impaired	92 (52.6)	40 (43.5)	52 (56.5)
Wall motion	Normal	170 (97.1)	81 (47.6)	89 (52.4)
	Abnormal	5(2.9)	2 (40.0)	3 (60.0)

ACEI: Angiotensin Converting Enzyme Inhibitors; CCB: Calcium Channel Blocker; ARB: Angiotensin Receptor Blocker; N: Numbers; %: Percentage; BMI: Body Mass Index; DM: Diabetes Mellitus; LVH: Left Ventricular Hypertrophy.

**Table 3:** Logistic regression for factors associated with microalbuminuria among newly diagnosed diabetic patients at Mulago hospital.

Factor		Microalbuminuria		Crude OR(CI).	P-value	Adjusted OR	P-value
		Absent No (%)	Present No (%)				
Sex	Male	44 (48.9)	46 (51.1)	1.00		1.00	
	Female	48 (56.5)	37 (43.5)	0.74 (0.41–1.34)	0.316	1.21 (0.26–5.70)	0.802
Age	< 40 years	25 (50.0)	25 (50.20)	1.00		1.00	
	40 and above	67 (53.6)	58 (46.4)	0.87 (0.45–1.67)	0.667	1.11 (0.24–5.16)	0.891
Pregnancy	No	46 (58.2)	33 (41.8)	1.00		1.00	
	Yes	1 (16.7)	5 (83.3)	6.84 (0.77–60.98)	0.085	7.74 (1.01–76.47)	0.050
Physical activity at work	Sedentary	6 (35.3)	11 (64.7)	1.00		1.00	
	Mild	25 (52.1)	23 (47.9)	0.47 (0.16–1.42)	0.183	0.08 (0.01–0.95)	0.046
	Moderate	38 (53.5)	33 (46.5)	0.37 (0.11–1.22)	0.104	0.07 (0.01–0.77)	0.030
	Strenuous	22 (59.5)	15 (40.5)	0.55 (0.03–1.37)	0.687	0.19 (0.13–2.73)	0.223
Physical activity at leisure	Sedentary	63 (51.2)	60 (48.8)	1.00			
	Moderate	29 (55.8)	23 (44.2)	0.83 (0.43–1.59)	0.582		
HbA1C	Normal	11 (78.6)	3 (21.4)	1.00		1.00	
	Abnormal	79 (50.0)	79 (50.0)	3.67 (0.99–13.65)	0.053	2.28 (0.32–16.16)	0.408
Hypertension	Normotensive	31 (46.3)	36 (53.7)	1.00		1.00	
	Hypertensive	61 (56.5)	47 (43.5)	0.66 (0.36–1.22)	0.189	2.38 (0.66–8.57)	0.183
Drugs	ACEI/ARBs	5 (55.6)	4 (44.4)	0.88 (0.23–3.40)	0.854		
	CCBs	9 (75.0)	3 (25.0)	0.35 (0.90–1.32)	0.121	0.38 (0.06–2.27)	0.289
	Beta blockers	2 (50.0)	2 (50.0)	1.11 (0.15–8.07)	0.917		
DM type	Type 1	16 (45.7)	19 (54.3)	1.00		1.00	
	Type 2	76 (54.3)	64 (45.7)	0.71 (0.34–1.49)	0.365	0.52 (0.08–3.56)	0.507
BMI	Normal weight	45 (43.7)	58 (56.3)	1.00		1.00	
	Over wt & Obesity	47 (65.3)	25 (34.7)	0.41 (0.22–0.77)	0.005	0.37 (0.12–1.13)	0.082
Ejection fraction	> 50%	74 (53.2)	65 (46.8)	1.00			
	< 50%	18 (50.0)	18 (50.0)	1.14 (0.55–2.37)	0.729		
LVH	Absent	36 (46.7)	41 (53.3)	1.00			
	Present	56 (57.1)	42 (42.9)	0.66 (0.36–1.20)	0.173		

OR: Odds Ratio; N: Number; %: Percentage; CI: Confidence Interval; BMI: Body Mass Index; DM: Diabetes Mellitus; LVH: Left Ventricular Hypertrophy; ACEI: Angiotensin Converting Enzyme Inhibitor; ARB: Angiotensin Receptor Blocker; CCB: Calcium Channel Blocker.

## Abbreviations

ACR: Albumin to Creatinine Ratio
ESRD: End Stage Renal Disease
DM: Diabetes Mellitus
CVD: Cardiovascular Diseases
LVH: Left Ventricular Hypertrophy
DN: Diabetic Nephropathy
BMI: Body Mass Index
SSA: Sub-Saharan Africa
